# Resectability and resection rates of colorectal liver metastases according to *RAS* and *BRAF* mutational status: prospective study

**DOI:** 10.1093/bjs/znac424

**Published:** 2022-12-13

**Authors:** Aki Uutela, Arno Nordin, Emerik Osterlund, Päivi Halonen, Raija Kallio, Leena-Maija Soveri, Tapio Salminen, Annika Ålgars, Ari Ristimäki, Ali Ovissi, Annamarja Lamminmäki, Timo Muhonen, Juha Kononen, Raija Ristamäki, Eetu Heervä, Hanna Stedt, Kaisa Lehtomäki, Soili Kytölä, Jari Sundström, Markus J Mäkinen, Lasse Nieminen, Teijo Kuopio, Mauri Keinänen, Pia Osterlund, Helena Isoniemi, the RAXO Study Group, Heikki Mäkisalo, Riikka Huuhtanen, Eila Lantto, Juhani Kosunen, Sirpa Leppä, Petri Bono, Johanna Mattson, Jari Räsänen, Anna Lepistö, Heidi Penttinen, Siru Mäkelä, Olli Carpén, Nina Lundbom, Antti Hakkarainen, Marjut Timonen, Veera Salminen, Niina Paunu, Irina Rinta-Kiikka, Martine Vornanen, Johanna Virtanen, Eija Korkeila, Eija Sutinen, Maija Lavonius, Jari Sundström, Roberto Blanco, Eija Pääkkö, Tiina Tuomisto-Huttunen, Päivi Auvinen, Vesa Kärjä, Sakari Kainulainen, Hannu-Pekka Kettunen, Ilmo Kellokumpu, Markku Aarnio, Ville Väyrynen, Kaija Vasala, Sanna Ketola, Kyösti Nuorva, Maija-Leena Murashev, Kalevi Pulkkanen, Venla Viitanen, Marko Nieppola, Elina Haalisto, Paul Nyandoto, Aino Aalto, Timo Ala-Luhtala, Jukka Tuominiemi, Anneli Sainast, Laura Pusa, Sanna Kosonen, Leena Helle, Terhi Hermansson, Riitta Kokko, Laura Aroviita, Petri Nokisalmi, Liisa Sailas, Heikki Tokola, Antti Jekunen, Teemu Pöytäkangas, Kari Möykkynen, Sanna Kosonen, Olli-Pekka Isokangas, Svea Vaarala, Tuula Klaavuniemi, Rainer Kolle, Peeter Karihtala, Mirja Heikkinen, Kaisu Johansson, Anna Sjöstrand, Piia Kajasviita, Jaana Kaleva-Kerola, Esa Männistö, Reneé Lindvall-Andersson, Tom Kaunismaa, Pia Vihinen, Nina Cavalli-Björkman

**Affiliations:** Department of Transplantation and Liver Surgery, Abdominal Centre, Helsinki University Hospital and University of Helsinki, Helsinki, Finland; Department of Transplant and Hepatopancreatobiliary Surgery, Royal Infirmary of Edinburgh, Edinburgh, UK; Department of Transplantation and Liver Surgery, Abdominal Centre, Helsinki University Hospital and University of Helsinki, Helsinki, Finland; Department of Transplantation and Liver Surgery, Abdominal Centre, Helsinki University Hospital and University of Helsinki, Helsinki, Finland; Department of Immunology, Genetics and Pathology, Uppsala University, Uppsala, Sweden; Department of Oncology, Helsinki University Hospital Comprehensive Cancer Centre and University of Helsinki, Helsinki, Finland; Department of Oncology, Oulu University Hospital, Oulu, Finland; Home Care Geriatric Clinic and Palliative Care, Joint Municipal Authority for Health Care and Social Services in Keski-Uusimaa, Hyvinkää, Finland; Faculty of Medicine, University of Helsinki, Helsinki, Finland; Department of Oncology, Tampere University Hospital and University of Tampere, Tampere, Finland; Department of Oncology, Turku University Hospital and University of Turku, Turku, Finland; Department of Pathology, HUS Diagnostic Centre and Applied Tumour Genomics, Research Programmes Unit, Helsinki University Hospital and University of Helsinki, Helsinki, Finland; Department of Radiology, HUS Medical Imaging Centre, Helsinki University Hospital and University of Helsinki, Helsinki, Finland; Department of Oncology, Kuopio University Hospital and University of Eastern Finland, Kuopio, Finland; Faculty of Medicine, University of Helsinki, Helsinki, Finland; Department of Oncology, South Carelia Central Hospital, Lappeenranta, Finland; Department of Oncology, Central Finland Hospital Nova, Jyväskylä, Finland; Docrates Cancer Center, Helsinki, Finland; Department of Oncology, Turku University Hospital and University of Turku, Turku, Finland; Department of Oncology, Turku University Hospital and University of Turku, Turku, Finland; Department of Oncology, Kuopio University Hospital and University of Eastern Finland, Kuopio, Finland; Department of Oncology, Tampere University Hospital and University of Tampere, Tampere, Finland; Department of Genetics, HUSLAB, HUS Diagnostic Centre, Helsinki University Hospital and University of Helsinki, Helsinki, Finland; Department of Pathology, Turku University Hospital and University of Turku, Turku, Finland; Department of Pathology, Oulu University Hospital and University of Oulu, Oulu, Finland; Department of Pathology, Tampere University Hospital and University of Tampere, Tampere, Finland; Department of Pathology, Central Finland Central Hospital, Jyväskylä, Finland; Department of Genetics, FIMLAB laboratories, Tampere University Hospital, Tampere, Finland; Department of Oncology, Helsinki University Hospital Comprehensive Cancer Centre and University of Helsinki, Helsinki, Finland; Department of Oncology, Tampere University Hospital and University of Tampere, Tampere, Finland; Department of Oncology/Pathology, Karolinska Institutet and Karolinska Sjukhuset, Cancer Centre of Excellence, Stockholm, Sweden; Department of Transplantation and Liver Surgery, Abdominal Centre, Helsinki University Hospital and University of Helsinki, Helsinki, Finland

## Introduction

Resection of colorectal liver metastases (CRLMs) improves survival and may lead to cure. Resectability rates can be improved with conversion therapy^1,2^.

*RAS* and *BRAF* mutations are found in 50 and 5–20 per cent of tumours respectively in patients with metastatic colorectal cancer^3,4^. These mutations limit systemic therapy alternatives^5^ and have been associated with worse outcomes in patients with CRLMs^6^. Patients with the *BRAF* V600E mutation clearly have shorter median survival, but some may survive without recurrence^7^.

Multidisciplinary teams (MDTs) have emerged to facilitate cooperation between medical specialties to ensure optimal care for the patient^8–11^. The aim of this study was to evaluate how *RAS* and *BRAF* mutational status affected resectability and conversion assessments performed by local hospitals and by a centralized MDT, and how this information could be used to improve resection rates and survival in patients with CRLMs.

## Methods

### Study design

RAXO was a prospective, investigator-initiated, nationwide Finnish study (NCT01531621, EudraCT 2011-003158-24) that included 1086 patients with metastatic colorectal cancer between 2012 and 2018. The main protocol^12^, liver metastases group^13^, and *RAS*/*BRAF* mutations in studies of metastatic colorectal cancer^14^ have been published previously. This substudy included patients with known *RAS/BRAF* status and CRLMs. Further details are available in the *supplementary material*. Patients with non-V600E *BRAF* mutations were excluded. The patients were assessed as having liver-only metastatic disease or liver and extrahepatic disease at the time of inclusion in the study. The central MDT at Helsinki University Hospital tertiary centre evaluated each patient’s technical resectability as described previously^12,13^ and in the *supplementary material*. The mutational status was mostly known to the local team, but only occasionally to the central MDT. Patients were classified into the following resection outcome groups: R0–1, R2/local ablative therapy (LAT) or systemic therapy only. The study was approved by the Ethics Committee at Helsinki University Hospital and all patients provided written informed consent. Statistical methodology is presented in the *supplementary material*.

## Results

Of 672 patients included, 226 (33.6 per cent) had *RAS*&*BRAF* wild-type (wt) tumours, 392 (58.3 per cent) *RAS* mutation (mt) tumours, and 54 (8.0 per cent) *BRAF*mt tumours. Median follow-up was 55 (95 per cent c.i. 50–59; minimum 18) months. Patient demographics are summarized in *Table S1*.

**Fig. 1:**
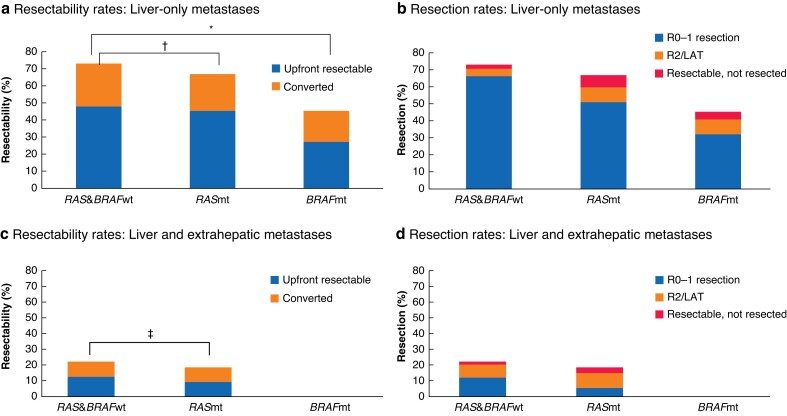
Resectability, conversion, and resection rates according to mutational status **a** Resectability and **b** resection rates for patients with liver-only metastases, and **c** resectability and **d** resection rates for patients with liver and extrahepatic metastases. wt, Wild type; mt, mutation; LAT, local ablative therapy. *OR 0.31 (95% c.i. 0.12 to 0.77); †OR 0.74 (0.46 to 1.22); ‡OR 0.79 (0.44 to 1.44).

Upfront resectability and conversion rates in the central assessment of 354 patients with liver-only and 318 with liver and extrahepatic metastases are shown in *Fig. 1*. In the liver-only group, the central MDT considered the metastases to be upfront resectable in 48.0, 45.5, and 27.3 per cent of patients with *RAS&BRAF*wt, *RAS*mt, and *BRAF*mt tumours respectively. Conversion rates for the borderline or unresectable liver-only group were 48.4, 39.5, and 25.0 per cent respectively. Conversion rates for patients with initially borderline liver-only CRLMs were 78.9 per cent for *RAS*&*BRAF*wt (reference), 81.5 per cent for *RAS*mt (OR 1.17, 95 per cent c.i. 0.42 to 3.32), and 40.0 per cent for *BRAF*mt (OR 0.18, 0.04 to 0.79) subgroups. The overall R0–1 resection rates for patients with liver-only CRLMs were 67.5 per cent for those with *RAS&BRAF*wt tumours (reference), 51.2 per cent for patients with *RAS*mt tumours (OR 0.51, 0.32 to 0.80), and 31.8 per cent for those with *BRAF*mt tumours (OR 0.22, 0.09 to 0.60). The influence of tumour location on conversion is shown in *Table S2*.

Patients with liver and extrahepatic *RAS&BRAF*wt and *RAS*mt metastases had similar upfront resectability and conversion rates. There was, however, a difference in R0–1 resection rates between the *RAS&BRAF*wt (12.6 per cent; reference) and *RAS*mt (4.9 per cent; OR 0.35, 0.15 to 0.87).

**Fig. 2:**
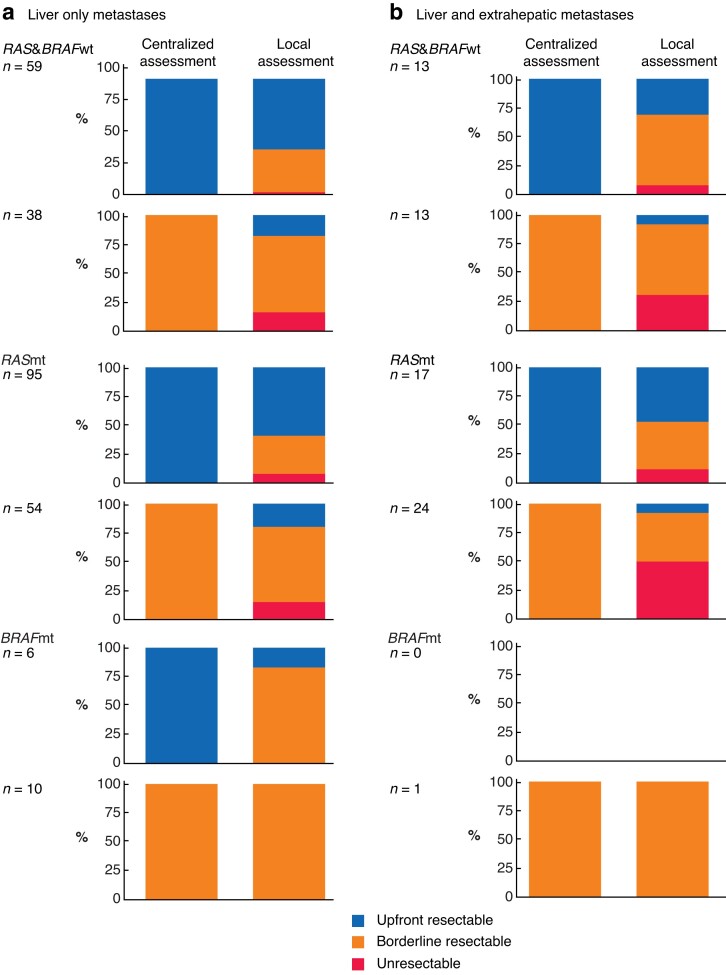
Centralized resectability assessment compared with local assessment according to mutational status for patients with liver-only metastases and those with liver and extrahepatic metastases **a** Patients with liver-only metastases and **b** patients with liver and extrahepatic metastases. wt, Wild type; mt, mutation.

When patients with liver-only disease were considered to have upfront resectable tumours by the central MDT, the local hospital underestimated resectability in 39, 41, and 83 per cent for *RAS&BRAF*wt, *RAS*mt, and *BRAF*mt tumours respectively (*Fig. 2a*). If the central MDT considered a patient to have borderline resectable disease, 16, 15, and 0 per cent respectively of the local assessments were scored as never resectable.

Among patients with liver and extrahepatic metastases considered upfront resectable by the central MDT, the local teams underestimated resectability in 69 and 53 per cent of those with *RAS&BRAF*wt and *RAS*mt tumours respectively (*Fig. 2b*). The rate of underestimation for borderline liver and extrahepatic metastases was 31 per cent for *RAS*&*BRAF*wt and 50 per cent for *RAS*mt tumours. Reasons for not resecting technically resectable metastases are listed in *Table S3*.

Forty-two patients (6.3 per cent) had CRLMs that the local team considered never resectable. These were considered upfront or borderline resectable in central assessment, and 28 became technically resectable. Nine of these patients underwent resection with curative intent, including six with *RAS&BRAF*wt tumours (5 with liver-limited and 1 with liver and extrahepatic metastases) and three with *RAS*mt tumours (2 liver-limited, and 1 liver and extrahepatic metastases).

Median overall survival (OS) after the first resection of metastases for 197 patients with liver-only metastases who underwent R0–1 resection was 82, 73, and 28 months according to *RAS*&*BRAF*wt (reference), *RAS*mt (HR 1.55, 0.91 to 2.65), and *BRAF*mt status (HR 7.24, 2.38 to 22.00) respectively (*P* < 0.001). Corresponding 5-year OS rates were 68, 60, and 0 per cent respectively (*Fig. S1*). For 22 patients with *RAS*&*BRAF*wt and *RAS*mt status who underwent R0–1 resection of liver and extrahepatic metastases, median OS was 79 and 71 months respectively (*P* = 0.847). Corresponding 5-year OS rates were 79 and 88 per cent. Recurrence-free survival, survival according to resection status, mutational status, and extent of disease, and a 12-month conditional landmark analysis of OS are shown in *Figs S2–S4*.

In multivariable analysis of risk factors for OS, assessment as unresectable by the central MDT appeared to be a strong risk factor. The second most notable factor was mutational status (*Table 1*).

**Table 1:** Multivariable analysis of risk factors for overall survival

	HR	
	Univariable analysis	Multivariable analysis
**Age > 70 years**	1.22 (1.01, 1.48)	1.27 (1.04, 1.56)
**Female sex**	1.04 (0.86, 1.26)	
**ECOG score**		
PS 0	1.00 (reference)	1.00 (reference)
PS 1	1.89 (1.50, 2.39)	1.47 (1.16, 1.87)
PS 2–3	3.38 (2.54, 4.48)	2.29 (1.70, 3.09)
**Charlson Co-morbidity Index score**		
0	1.00 (reference)	
1–2	1.20 (0.96, 1.48)	
3–5	1.25 (0.47, 3.36)	
**BMI (kg/m^2^)**		
< 20	1.00 (reference)	
20–30	0.96 (0.67, 1.36)	
> 30	0.85 (0.57, 1.27)	
Primary tumour in right colon	1.76 (1.44, 2.14)	1.76 (1.42, 2.18)
Primary tumour not operated at baseline (yes *versus* no)	1.89 (1.53, 2.27)	1.49 (1.20, 1.85)
Synchronous metastases*	1.10 (1.20, 1.88)	1.35 (1.04, 1.77)
≥ 3 liver segments involved	2.37 (1.91, 2.94)	1.54 (1.21, 1.98)
Extrahepatic metastases	2.41 (2.00, 2.91)	1.21 (0.97, 1.50)
**Mutational status**		
*RAS* and *BRAF* wild type	1.00 (reference)	1.00 (reference)
*RAS* mutation	1.57 (1.27, 1.93)	1.62 (1.30, 2.00)
*BRAF* mutation	3.34 (3.09, 6.07)	2.55 (1.78, 3.64)
**Upfront resectability assessment by central MDT**		
Resectable	1.00 (reference)	1.00 (reference)
Borderline	1.50 (1.09, 2.06)	1.11 (0.79, 1.55)
Unresectable	5.54 (4.28, 7.18)	3.69 (2.68, 5.08)

Values in parentheses are 95% confidence intervals. Co-variables significant in univariable analyses were entered into the multivariable analysis. *Within 2 months of diagnosis of primary tumour. ECOG, Eastern Cooperative Oncology Group; PS, performance status; MDT, multidisciplinary team.

## Discussion

With the help of centralized multidisciplinary assessment, high resectability, conversion, and resection rates are achievable for patients with *RAS*&*BRAF*wt and *RAS*mt CRLMs. Selected patients with unfavourable *BRAF* mutation or with extrahepatic metastases may even undergo potentially curative resection.

Prospective studies of highly selected patients, often with *RAS*wt tumours, have reported a conversion rate of 44–64 per cent and secondary resection/LAT rates of 44–61 per cent for patients with initially borderline or unresectable CRLMs^2,15–17^. In patients who also underwent hepatic artery infusion as induction therapy, conversion and secondary resection/LAT rates were 32 per cent for patients with *RAS*&*BRAF*wt tumours, 39 per cent for those with *RAS*mt lesions, and 0 per cent for those with *BRAF*mt disease^18^. In a retrospective neoadjuvant therapy response assessment^19^, disease in up to 53 per cent of patients, mostly with *RAS*wt tumours, was considered resectable upfront or after conversion, but only 29 per cent of these patients actually underwent resection. The present prospective study has shown comparable secondary resection/LAT rates for patients with liver-only *RAS*&*BRAF*wt and *RAS*mt metastases, and a secondary resection/LAT rate as high as 25 per cent for patients with *BRAF*mt tumours.

In the literature, resectability and resection rates range from 18 to 71 per cent and from 16 to 54 per cent respectively for patients with liver-only metastases^1,9,19,20^. This study has shown that the chance of curative resection is highest for liver-only *RAS*&*BRAF*wt metastases, then *RAS*mt metastases. Even for patients with tumours harbouring a *BRAF* mutation, a resectability rate of 45 per cent and corresponding resection rate of 32 per cent provided at least a chance of prolonged survival.

Other groups have reported up to 44 per cent resectability, but resection rates of only 5–11 per cent for patients with multiorgan metastases^19,20^. The present study has shown that such patients are indeed less likely to undergo resection. After conversion therapy, curative resection of all diseased organs could still be completed in 13 and 5 per cent of patients with *RAS&BRAF*wt and *RAS*mt tumours respectively.

Reported disagreements between surgeons assessing resectability of 35–52 per cent, including 7–11 per cent major disagreements, have stressed the importance of a multidisciplinary team^9,15^. Disagreement between local teams and central MDT was considerable in the present study, with most major disagreements relating to borderline resectable or extrahepatic disease, suggesting that patients with more advanced disease could benefit even more from centralized MDT assessment. In multivariable survival analysis, central MDT assessment was associated with survival. This indicates that the central MDT is capable of including important clinical and radiological information in their decision, and underlines the potential additional value of multidisciplinary assessment of patients with liver-only or liver-dominant CRLMs.

## Collaborators

RAXO Study Group: Heikki Mäkisalo, Riikka Huuhtanen, Eila Lantto, Juhani Kosunen, Sirpa Leppä, Petri Bono, Johanna Mattson, Jari Räsänen, Anna Lepistö, Heidi Penttinen, Siru Mäkelä, Olli Carpén, Nina Lundbom, Antti Hakkarainen, Marjut Timonen (Helsinki University Hospital, Helsinki, Finland); Veera Salminen, Niina Paunu, Irina Rinta-Kiikka, Martine Vornanen (Tampere University Hospital, Tampere, Finland); Johanna Virtanen, Eija Korkeila, Eija Sutinen, Maija Lavonius, Jari Sundström, Roberto Blanco (Turku University Hospital, Turku, Finland); Eija Pääkkö (Oulu University Hospital, Oulu, Finland); Tiina Tuomisto-Huttunen, Päivi Auvinen, Vesa Kärjä, Sakari Kainulainen, Hannu-Pekka Kettunen (Kuopio University Hospital, Kuopio, Finland); Ilmo Kellokumpu, Markku Aarnio, Ville Väyrynen, Kaija Vasala, Sanna Ketola, Kyösti Nuorva (Central Finland Hospital Nova, Jyväskylä, Finland); Maija-Leena Murashev, Kalevi Pulkkanen, Venla Viitanen, Marko Nieppola, Elina Haalisto (Satakunta Central Hospital, Pori, Finland); Paul Nyandoto, Aino Aalto (Päijät-Häme Central Hospital, Lahti, Finland); Timo Ala-Luhtala, Jukka Tuominiemi (Seinäjöki Central Hospital, Seinäjoki, Finland), Anneli Sainast, Laura Pusa, Sanna Kosonen, Leena Helle (Kymenlaakso Central Hospital, Kotka, Finland); Terhi Hermansson (Kymenlaakso Central Hospital, Kotka, Finland and South Savo Central Hospital, Mikkeli, Finland); Riitta Kokko, Laura Aroviita, Petri Nokisalmi (Kanta-Häme Central Hospital, Hämeenlinna, Finland); Liisa Sailas, Heikki Tokola (North Karelia Central Hospital, Joensuu, Finland); Antti Jekunen, Teemu Pöytäkangas (Vaasa Central Hospital, Vaasa, Finland); Kari Möykkynen, Sanna Kosonen (South Karelia Central Hospital, Lappeenranta, Finland); Olli-Pekka Isokangas, Svea Vaarala (Lapland Central Hospital, Rovaniemi, Finland); Tuula Klaavuniemi, Rainer Kolle (South Savo Central Hospital, Mikkeli, Finland); Peeter Karihtala, Mirja Heikkinen (Kainuu Central Hospital, Kajaani, Finland); Kaisu Johansson, Anna Sjöstrand, Piia Kajasviita (Central Ostrobothnia Central Hospital, Kokkola, Finland); Jaana Kaleva-Kerola (Länsi-Pohja Central Hospital, Kemi, Finland); Esa Männistö (Savonlinna Central Hospital, Savonlinna, Finland); Reneé Lindvall-Andersson, Tom Kaunismaa, Pia Vihinen, Nina Cavalli-Björkman (Åland Central Hospital, Mariehamn, Finland).

## Supplementary Material

znac424_Supplementary_Data

## Data Availability

The original study database is not publicly available, but an anonymized version of the original data can be requested from the authors.
