# Supplementary material for: Resectability and resection rates of colorectal liver metastases according to RAS and BRAF mutational status: prospective study
Source: Br J Surg. 2022 Dec 13;110(8):931–5. doi: 10.1093/bjs/znac424 (PMC10361677; doi:10.1093/bjs/znac424)
Supplement: znac424_Supplementary_Data [file znac424_supplementary_data.docx]

**Liver and extrahepatic resectability, resection rates, and survival with centralized assessment of patients with colorectal liver metastases according to *RAS* and *BRAF* mutational status in a prospective study**

**Authors**

1. Aki Uutela, MD^1,2^
2. Arno Nordin, MD PhD*^1^
3. Emerik Osterlund, MD*^1,3^
4. Päivi Halonen, MD PhD^4^
5. Raija Kallio, MD PhD^5^
6. Leena-Maija Soveri, MD PhD^6,7^
7. Tapio Salminen, MD^8^
8. Annika Ålgars, MD PhD^9^
9. Ari Ristimäki, MD Prof^10^
10. Ali Ovissi, MD^11^
11. Annamarja Lamminmäki, MD PhD^12^
12. Timo Muhonen, MD, PhD^7, 13^
13. Juha Kononen, MD, PhD^14, 15^
14. Raija Ristamäki, MD PhD^9^
15. Eetu Heervä, MD PhD^9^
16. Hanna Stedt, MD PhD^12^
17. Kaisa Lehtomäki, MD^8^
18. Soili Kytölä, MSc PhD^16^
19. Jari Sundström, MD PhD^17^
20. Markus J Mäkinen, MD Prof^18^
21. Lasse Nieminen, MD^19^
22. Teijo Kuopio, MD PhD^20^
23. Mauri Keinänen, MSc PhD^21^
24. Pia Osterlund, MD Prof**^4,8,22^
25. Helena Isoniemi, MD Prof**^1^

on behalf of the RAXO Study Group

* shared second authorship

** shared last authorship

*^1^ Department of Transplantation and Liver Surgery, Abdominal Center, Helsinki University Hospital and University of Helsinki, Helsinki, Finland*

*^2^ Department of Transplant and HPB Surgery, Royal Infirmary of Edinburgh, Edinburgh, United Kingdom*

*^3^ Department of Immunology, Genetics and Pathology, Uppsala University, Uppsala, Sweden*

*^4^ Department of Oncology, Helsinki University Hospital Comprehensive Cancer Center and University of Helsinki, Helsinki, Finland*

*^5^ Department of Oncology, Oulu University Hospital, Oulu, Finland*

*^6^ Home Care Geriatric Clinic and Palliative Care, Joint Municipal Authority for Health Care and Social Services in Keski-Uusimaa, Hyvinkää, Finland*

*^7^ Faculty of Medicine, University of Helsinki, Helsinki, Finland*

*^8^ Department of Oncology, Tampere University Hospital and University of Tampere, Tampere, Finland*

*^9^ Department of Oncology, Turku University Hospital and University of Turku, Turku, Finland*

*^10^ Department of Pathology, HUS Diagnostic Centre and Applied Tumour Genomics, Research Programs Unit, Helsinki University Hospital and University of Helsinki, Helsinki, Finland*

*^11^Department of Radiology, HUS Medical Imaging Centre, Helsinki University Hospital and University of Helsinki, Helsinki, Finland*

*^12^ Department of Oncology, Kuopio University Hospital and University of Eastern Finland, Kuopio, Finland*

*^13^ Department of Oncology, South Carelia Central Hospital, Lappeenranta, Finland*

*^14^ Department of Oncology, Central Finland Hospital Nova, Jyväskylä, Finland*

*^15^ Docrates Cancer Center, Helsinki, Finland*

*^16^ Department of Genetics, HUSLAB, HUS Diagnostic Center, Helsinki University Hospital and University of Helsinki, Helsinki, Finland*

*^17^ Department of Pathology, Turku University Hospital and University of Turku, Turku, Finland*

*^18^ Department of Pathology, Oulu University Hospital and University of Oulu, Oulu, Finland*

*^19^ Department of Pathology, Tampere University Hospital and University of Tampere, Tampere, Finland*

*^20^ Department of Pathology, Central Finland Central Hospital, Jyväskylä, Finland*

*^21^ Department of Genetics, FIMLAB laboratories, Tampere University Hospital, Tampere, Finland.*

*^22^ Department of Oncology/Pathology, Karolinska Institutet and Karolinska sjukhuset, Cancer centre of excellence, Stockholm, Sweden*

Corresponding author

Aki Uutela, MD

Transplantation and Liver Surgery

Abdominal Center,

Helsinki University Hospital and University of Helsinki, Helsinki, Finland

Transplantation office, Haartmaninkatu 4, building 1, 00029 HUS, Finland

Phone: +358 50 512 3529

E-mail: aki.uutela@hus.fi

ORCID identifier: 0000-0003-1324-5894

Twitter: @Aki_Uutela

## The RAXO Study Group Investigators

*Helsinki University Hospital*

Pia Österlund, Helena Isoniemi, Aki Uutela, Leena-Maija Soveri, Päivi Halonen, Arno Nordin, Heikki Mäkisalo, Riikka Huuhtanen, Eila Lantto, Ali Ovissi, Juhani Kosunen, Sirpa Leppä, Petri Bono, Johanna Mattson, Jari Räsänen, Anna Lepistö, Emerik Österlund, Heidi Penttinen, Siru Mäkelä, Ari Ristimäki, Olli Carpén, Nina Lundbom, Antti Hakkarainen, Marjut Timonen.

*Tampere University Hospital*

Tapio Salminen, Pia Österlund, Kaisa Lehtomäki, Veera Salminen, Niina Paunu, Irina Rinta-Kiikka, Martine Vornanen, Nieminen Lasse.

*Turku University Hospital*

Annika Ålgars, Raija Ristamäki, Eetu Heervä, Johanna Virtanen, Eija Korkeila, Eija Sutinen, Maija Lavonius, Jari Sundström, Roberto Blanco

*Oulu University Hospital*

Raija Kallio, Markus Mäkinen, Eija Pääkkö

*Kuopio University Hospital*

Annamarja Lamminmäki, Hanna Stedt, Tiina Tuomisto-Huttunen, Päivi Auvinen, Vesa Kärjä, Sakari Kainulainen, Hannu-Pekka Kettunen

*Central Finland Hospital Nova*

Ilmo Kellokumpu, Markku Aarnio, Ville Väyrynen, Kaija Vasala, Juha Kononen, Sanna Ketola, Teijo Kuopio, Kyösti Nuorva

*Satakunta Central Hospital*

Pia Österlund, Maija-Leena Murashev, Kalevi Pulkkanen, Venla Viitanen, Marko Nieppola, Elina Haalisto

*Päijät-Häme Central Hospital*

Paul Nyandoto, Aino Aalto

*Seinäjoki Central Hospital*

Timo Ala-Luhtala, Jukka Tuominiemi

*Kymenlaakso Central Hospital*

Anneli Sainast, Timo Muhonen, Laura Pusa, Sanna Kosonen, Leena Helle, Terhi Hermansson

*Kanta-Häme Central Hospital*

Riitta Kokko, Laura Aroviita, Petri Nokisalmi

*North Karelia Central Hospital*

Liisa Sailas, Heikki Tokola

*Vaasa Central Hospital*

Antti Jekunen, Teemu Pöytäkangas

*South Carelia Central Hospital*

Kari Möykkynen, Sanna Kosonen, Timo Muhonen

*Lapland Central Hospital*

Olli-Pekka Isokangas, Svea Vaarala

*South Savo Central Hospital*

Terhi Hermansson, Tuula Klaavuniemi, Rainer Kolle

*Kainuu Central Hospital*

Raija Kallio, Peeter Karihtala, Mirja Heikkinen

*Central Ostrobothnia Central Hospital*

Kaisu Johansson, Anna Sjöstrand, Piia Kajasviita

*Länsi-Pohja central hospital*

Jaana Kaleva-Kerola

*Savonlinna Central Hospital*

Esa Männistö

*Åland Central Hospital*

Reneé Lindvall-Andersson, Tom Kaunismaa, Pia Vihinen, Nina Cavalli-Björkman

## Supplementary Materials - Index

| Supplementary Methods |  | |
| --- | --- | --- |
| Study design | *page 7* | |
| Systemic therapy | *page 7* | |
| Molecular pathology | *page 7* | |
| Resectability assessment | *page 7* | |
| Treatment groups | *page 8* | |
| Statistical analyses | *page 8* | |
| Supplementary Results | *page. 10* | |
| **Strengths** and Limitations | *page 12* | |
| Supplementary Figures and Tables |  | |
| Supplementary figure 1. Survival from first R0-1 resection | | *page 13* |
| Supplementary figure 2. Recurrence free survival (RFS) | | *page 14* |
| Supplementary figure 3. Survival of patients according to resection status | | *page 15* |
| Supplementary figure 4. 12-month conditional Landmark analysis of overall survival of patients according to resection status | | *page 16* |
| Supplementary table 1. Patient demographics | | *page 17* |
| Supplementary table 2. Conversion rates for initially borderline resectable patients by primary tumour sidedness and systemic therapy regimens | | *page 19* |
| Supplementary table 3. Reasons for not resecting patients who were centrally assessed as technically resectable | | *page 20* |
| References | | *page 21* |

## Supplementary Methods

### Study design

The whole RAXO study population included 1086 patients, from which we excluded the patients with unknown *KRAS, NRAS* or *BRAF* status (n=155), who did not have liver metastases at diagnosis of mCRC (n=240), who only received best supportive care (n=14), or who had an atypical *BRAF* (non-*V600E*) mutation (n=5). The remaining 672 patients formed the cohort for this substudy. The single patient-level clinical data of these patients was collected to centrally evaluated and monitored databases as described earlier.^1-3^

### Systemic therapy

Standard local systemic therapy treatment protocols based on ESMO^4^ and NCCN,^5, 6^ guidelines were used to choose first-line systemic therapy, given until disease progression, toxicity, or resectability was achieved. In the neoadjuvant setting, oxaliplatin-based treatment was used.^7^ For conversion chemotherapy, the most intensive regimen, presumed tolerable, was used, mostly a doublet or triplet chemotherapy combined with a targeted agent (bevacizumab, cetuximab, or panitumumab) based on *RAS* and *BRAF* status.^4^

### Molecular pathology

*KRAS*, *NRAS,* and *BRAF*^V600E^ mutations were analysed with reverse transcriptase polymerase chain reaction (PCR) in 49%, next generation sequencing (NGS) in 38%, Idylla panels in 13%, and Sanger sequencing or pyrosequencing in <1%, from either histological biopsy or resection specimen from the primary tumour or liver metastasis. The *RAS*&*BRAF*wt samples were tested for at least *KRAS* and *NRAS* exons 2-4 and *BRAF*^V600E^. Of the *RAS*mt patients, 222 were not analysed for *BRAF* but were assumed to be *BRAF*wt, as the coexistence of *RAS* and *BRAF* mutations was considered rare.^8^ Immunohistochemistry with primary antibodies for MLH1, MSH2, MSH6, and PMS2 proteins was used to identify deficient mismatch repair status (dMMR), and PCR was used when the results of immunohistochemistry were indeterminate.

### Resectability assessment

The first local resectability assessment was performed at the local hospital before recruitment of the patient for the RAXO study. The radiological data used in the local assessment was sent to the centralized MDT. Imaging examinations consisted of at least contrast-enhanced whole body (chest, abdomen, and pelvis) computed tomography (CT) and were supplemented as required by magnetic resonance imaging and 18F-fluoro-deoxyglucose positron emission tomography. The MDT consisted of experts in liver surgery and abdominal radiology with medical oncologists, radiation oncologists, colorectal, thoracic, and cytoreductive surgeons, gynaecologists, thoracic radiologists, PET/CT specialists, and pathologists as required. The main factors considered for resectability were number, size and localization of metastases, including any possible contact or invasion of vital structures such as main hepatic vessels and bile ducts. The MDT assessment was performed at baseline and repeated twice every 2-3 months of systemic therapy. The final treatment decisions were made by local MDTs and the resections were either carried out locally or the patient was referred to the tertiary unit.

### Treatment groups

The whole RAXO database includes all metastasectomies of any organ. In many extrahepatic metastases, resection margins between R0 and R1 were difficult to determine, especially for peritoneal metastasis. Furthermore, initially, structured pathology reports were not harmonized at all hospitals. Therefore, the R0 and R1 resections were combined, and the term R0-1 resection or curative resection is used. As the microscopic completeness of LAT cannot be verified, these were grouped together with macroscopically incomplete R2 resections, or those with a second organ not resected, denoted R2/LAT. The third group consisted of patients who were never resected or treated with LAT and received systemic therapy; ‘systemic therapy only’.

### Statistical analyses

The Bonferroni corrected Chi-square was used to calculate differences in demographics. Logistic regression was used to calculate odds ratios (OR) with 95 per cent confidence intervals (95% CI) for demographic variables with significant differences. The Kaplan-Meier method with log rank statistics was used to calculate overall survival (OS) and recurrence-free survival (RFS), which were calculated separately from the first resection and from the diagnosis of metastatic disease to the date of death or censored at last follow-up. A conditional 12-month Landmark analysis of OS was used to control a potential guarantee-time bias as reported previously.^1^ Hazard ratios (HR) and corresponding 95% CIs for survival were calculated using Cox proportional hazard regression. Univariable analyses were first performed with potential prognostic covariates, and those with significant HR were then entered in a multivariable analysis. The study had 460 OS events in 672 patients, which allowed for several covariates in multivariable analysis. The median follow-up time was calculated with the reverse Kaplan-Meier method. The RAXO study database is prospective and collected separately for each patient. Patients with missing *RAS*/*BRAF* data were excluded from the study as described above. MMR analyses were not available for 68 per cent of the patients, and MMR status was not included in survival analysis. Otherwise, the analysed data was complete for all patients. SPSS Statistics, Version 25.0, Armonk, NY, was used. The data cut-off date for follow-up was March 27, 2020.

## Supplementary Results

There were 354 patients (53 per cent) in the ‘liver-only’ group, of which 123 (35 per cent) were *RAS*&*BRAF*wt, 209 (59 per cent) were *RAS*mt and 22 (6 per cent) were *BRAF*mt patients. The ‘liver and extrahepatic’ group included 318 patients (47 per cent), of which 103 (32 per cent) were *RAS*&*BRAF*wt, 183 (58 per cent) were *RAS*mt, and 32 (10 per cent) were *BRAF*mt. There were no significant differences in mutation frequencies between these groups. MMR testing was available for 212 (32 per cent) patients, and there were five dMMRs, three *RAS*mt and two *BRAF*mt. By the end of the follow-up, 460 of the patients (69 per cent) were deceased, mostly due to progressive metastatic CRC. No patients were lost to follow-up.

*RAS&BRAF*wt/*RAS*mt/*BRAF*mt groups showed significant differences in sex (men 71/62/44 per cent), location of primary (right colon 14/30/69 per cent, left colon 50/36/20 per cent, rectum 36/34/9 per cent), peritoneal metastases (7/8/22 per cent), and distant lymph node metastases (25/18/37 per cent), respectively ([Supplementary Table 1](#_Supplementary_table_1.)).

For borderline patients with ‘liver-only’ metastases and left-sided primary tumours (left-sided colon or rectum), conversion rates were 81 per cent in *RAS*&*BRAF*wt, 86 per cent in *RAS*mt, and 100 per cent in *BRAF*mt, and for patients with right-sided primaries they were 0 per cent (one patient) in *RAS*&*BRAF*wt, 71 per cent in *RAS*mt, and 14 per cent in *BRAF*mt ([Supplementary table 2](#_Supplementary_table_2.)). Corresponding conversion rates for patients with ‘liver and extrahepatic’ for *RAS*&*BRAF*wt/*RAS*mt/*BRAF*mt were 100/67/0 per cent for patients with left-sided primary and 50/60/- per cent for patients with right-sided primary.

For the group with ‘liver-only’ metastases, conversion rates for borderline resectable patients with left-sided (left colon or rectum) primaries were 90 per cent with doublet chemotherapy and cetuximab/panitumumab, 88 per cent with doublet/triplet chemotherapy and bevacizumab, and 69 per cent with 1 or 2 drugs (1 or 2 cytotoxics +/- biologic). With ‘liver-only’ metastatic right-sided primary colon cancers, conversion rates were 0 per cent with cetuximab/panitumumab-based, 55 per cent with bevacizumab-based, and 50 per cent with 1 or 2 drugs. In case of ‘liver and extrahepatic’ metastases, conversion rates for borderline resectable left-sided primary tumours were 67 per cent with doublet and cetuximab/panitumumab, 60 per cent with doublet/triplet chemotherapy and bevacizumab, and 33 per cent with 1 or 2 drugs. For ‘liver and extrahepatic’ patients with right colon primaries, the corresponding conversion rates were 63 per cent for doublet/triplet chemotherapy and bevacizumab and 67 per cent for 1 or 2 drugs. Patients with ‘liver-only’ metastases and left-sided primary had a higher chance of conversion compared to patients with right-sided primary (OR 5.00 [1.84-13.56]). This difference was not seen in patients with ‘liver and extrahepatic’ metastases (OR 0.71 [0.17-3.03]).

There were 19 patients with ‘liver-only’ and 9 with ‘liver and extrahepatic’ metastases, who were centrally assessed as technically resectable, but were not resected ([Supplementary table 3](#_Supplementary_table_3.)). The most common reason for non-operative approach was progressive disease for 12 patients, others were comorbidities for 5, and inoperable status discovered at exploratory laparotomy for 2.

For ‘liver-only’ patients 1-year RFS-rates after R0-1 resection were 64/58/29 per cent for *RAS*&*BRAF*wt/*RAS*mt/*BRAF*mt, respectively, HR ref./1.14 (0.79-1.63)/2.41 (1.03-5.66), p=0.112 ([Supplementary figure 2](#_Supplementary_figure_1.)). For patients with ‘liver and extrahepatic’ disease, 1-year RFS-rates were 62/33/- per cent, respectively, HR ref./2.15 (0.82-5.60)/-, p=0.11.

From the diagnosis of metastatic disease, mOS for R0-1-resected patients with ‘liver-only’ metastases was 83/75/30 months for *RAS*&*BRAF*wt/*RAS*mt/*BRAF*mt, respectively, while ‘liver-only’ patients with R2 resection/LAT had mOS of not reached/37/16 months, and ‘systemic therapy only’ patients’ mOS were 27/19/19 months, respectively ([Supplementary figure 3](#_Supplementary_figure_2.)). R0-1-resected patients with ‘liver and extrahepatic’ metastases had mOS of 86/71/- months from diagnosis of metastases. Patients with R2 resection or LAT had mOS of 42/50/- months, and systemic therapy only patients had mOS of 25/21/11 months. A 12-month conditional Landmark analysis of OS yielded similar results ([Supplementary figure 4](#_Supplementary_figure_4.)).

In univariable analysis, right-sided primary tumour was a negative prognostic factor for OS for *RAS*&*BRAF*wt patients with ‘liver-only’ metastases (HR 2.16 [95%CI 1.01-4-63]), but this was not as clearly seen in *RAS*mt (HR 1.43 [0.91-2.07]) or *BRAF*mt (HR 2.24 [0.70-7.17]) groups. The trend was same for patients with ‘liver and extrahepatic’ metastases with HR 1.71 (0.99-2.95)/1.13 (0.80-1.60)/1.24 (0.55-2.81) for *RAS*&*BRAF*wt/*RAS*mt/*BRAF*mt, respectively. Both local and central MDT assessment results associated with survival in univariate analyses. In addition to resectability and mutational status, poor ECOG performance status, right-sided primary tumour, primary tumour not operated, synchronous appearance of metastases, metastases in three or more liver segments, and age over 70 years were also associated with shorter OS in multivariable analysis (Table 1 in main publication).

## Strengths and limitations

The strength of this study is the prospective multicentre analysis of 672 treatable patients with CRC liver metastases and known *RAS* and *BRAF* status. The single patient-level clinical data of these patients was collected to centrally evaluated and monitored databases as described earlier.^1, 2^ The repeated centralized multidisciplinary resectability assessment was done at the Finnish national tertiary liver centre to provide state-of-the-art treatment recommendations for all of the patients with liver metastases by optimising resectability, conversion, and resection. *RAS* and *BRAF* status were known for 83 per cent of RAXO patients with liver metastases and this group was very similar to the main study population. The mutational analyses were mainly performed during the clinical workup at the local hospitals, and their results were reflected in the choice of systemic therapies. As the mutational status was mostly not known in centralized MDT assessment, the technical resectability assessments was not affected by mutations. This reduced the risk of restricting a technically resectable patient from being offered potentially curative treatment. To our knowledge, this kind of MDT assessment data combined with mutational status has not been presented before.

There are certain limitations inherent to the study design. The RAXO study was intended as a nation-wide non-randomized study with centralized MDT assessment as the intervention. During the recruitment period, treatment guidelines were updated regarding sidedness and extended *RAS* and *BRAF* testing, which affected the number but not the outcome of patients included in this sub-study as all were fully tested.^4-6^ An observational study can never control the confounding factors as well as a strictly defined randomised study, but rather reflects the real-world setting and a different patient population. Another limitation is the small number of resected *BRAF*mt patients. This shortcoming is common to most prospective studies due to relative rarity and aggressive nature of the mutation. Collaborative registries and international prospective studies are required to define more precisely which *BRAF*mt patients benefit most from liver resection.

## Supplementary Figures and Tables

## Supplementary figure 1. Survival from first R0-1 resection A. Patients with ‘liver-only’ metastases, p<0.001 B. Patients with ‘liver and extrahepatic’ metastases, p=0.847.


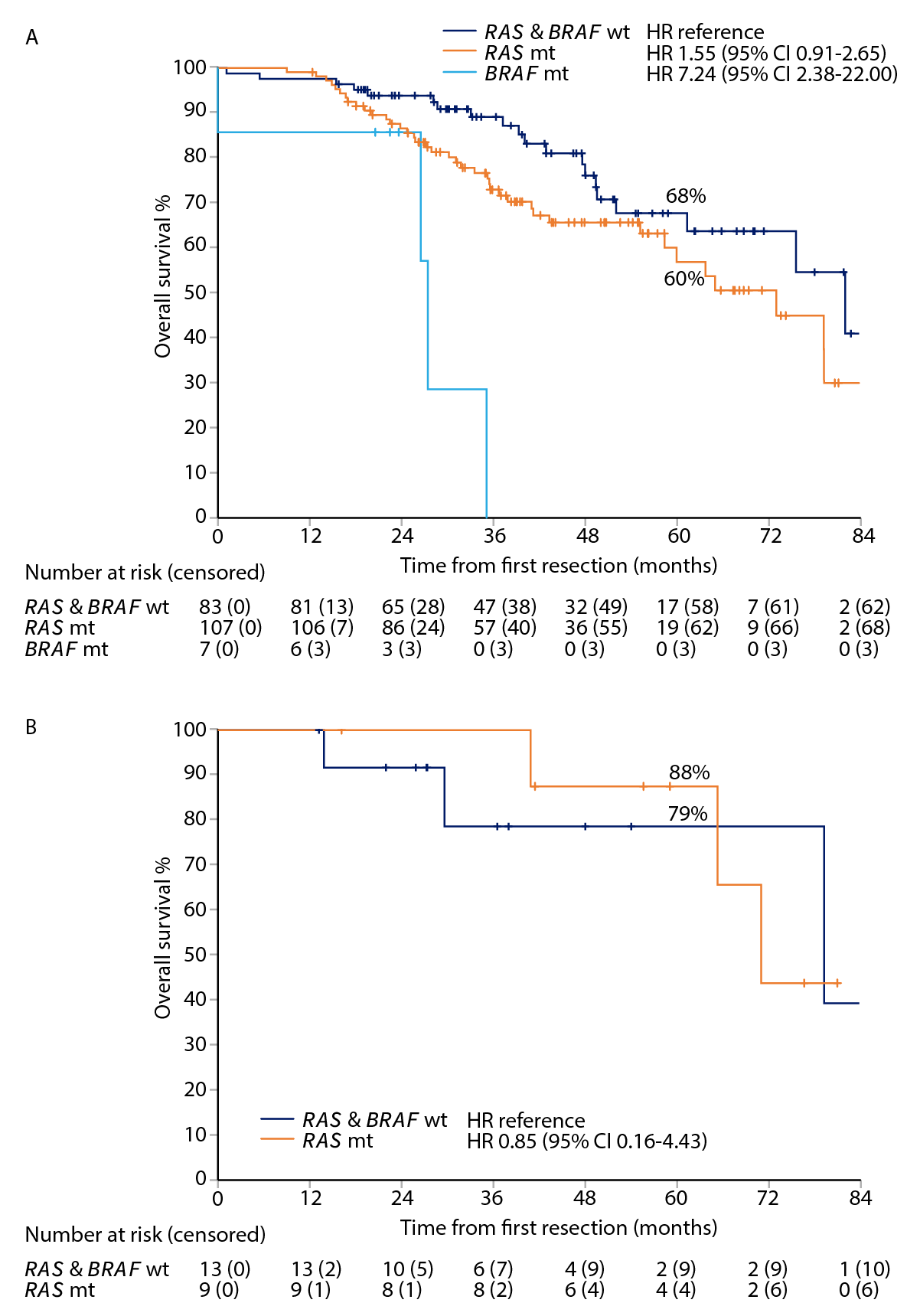


## Supplementary figure 2. Recurrence free survival (RFS) after complete macroscopic resection of metastatic disease, including all metastatic sites and the primary tumour A. Patients with initially ‘liver-only’ metastatic disease B. Patients with ‘liver and extrahepatic’ metastases at presentation of metastatic disease


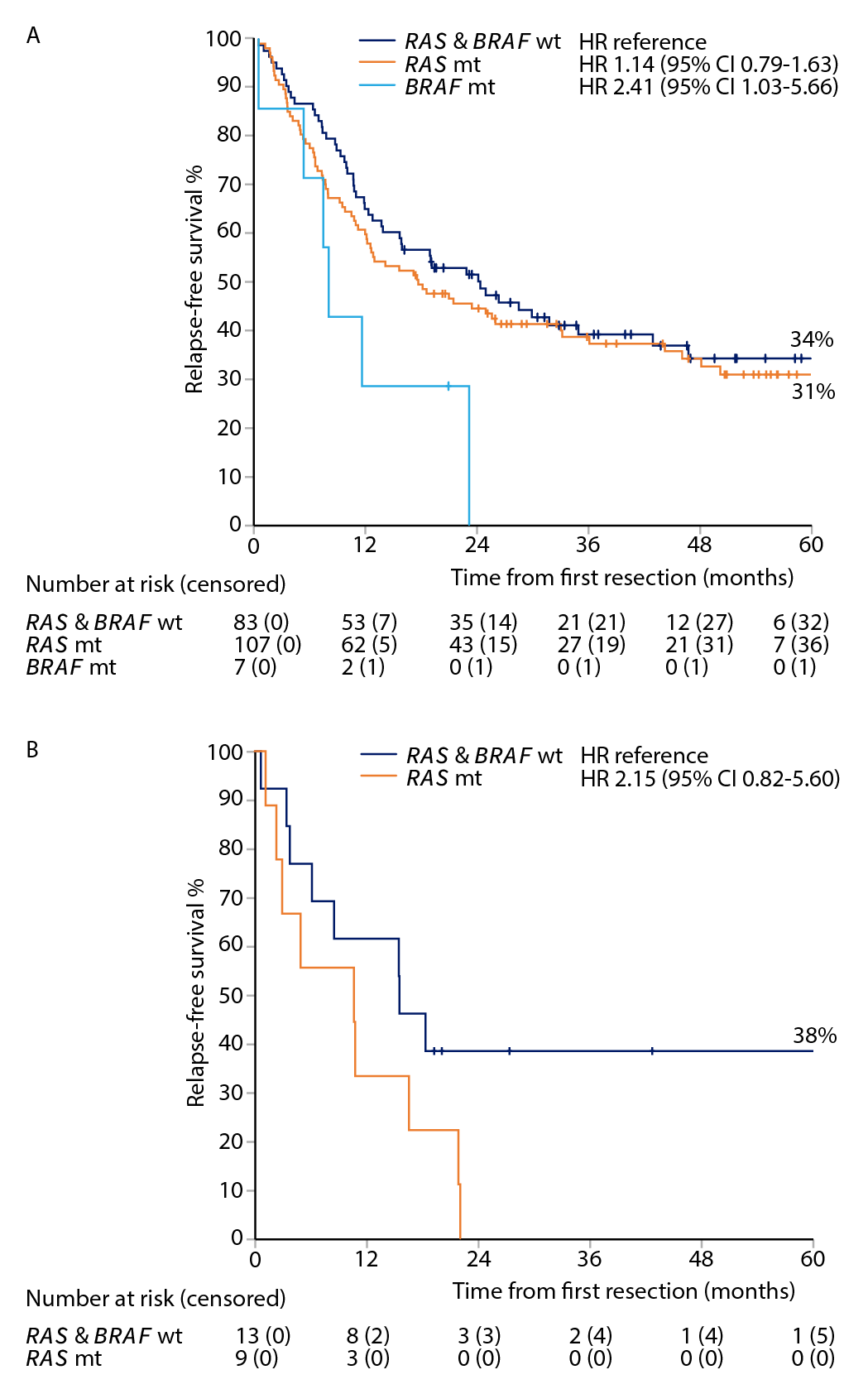


## Supplementary figure 3. Survival of patients according to resection status (R0/R1, R2 or LAT, systemic therapy only), mutations, and extent of disease A. *RAS&BRAF*wt ‘liver-only’ B. *RAS*mt ‘liver-only’ C. *BRAF*mt ‘liver-only’ D. *RAS&BRAF*wt ‘liver and extrahepatic’ E. *RAS*mt ‘liver and extrahepatic’. F. *BRAF*mt ‘liver and extrahepatic’


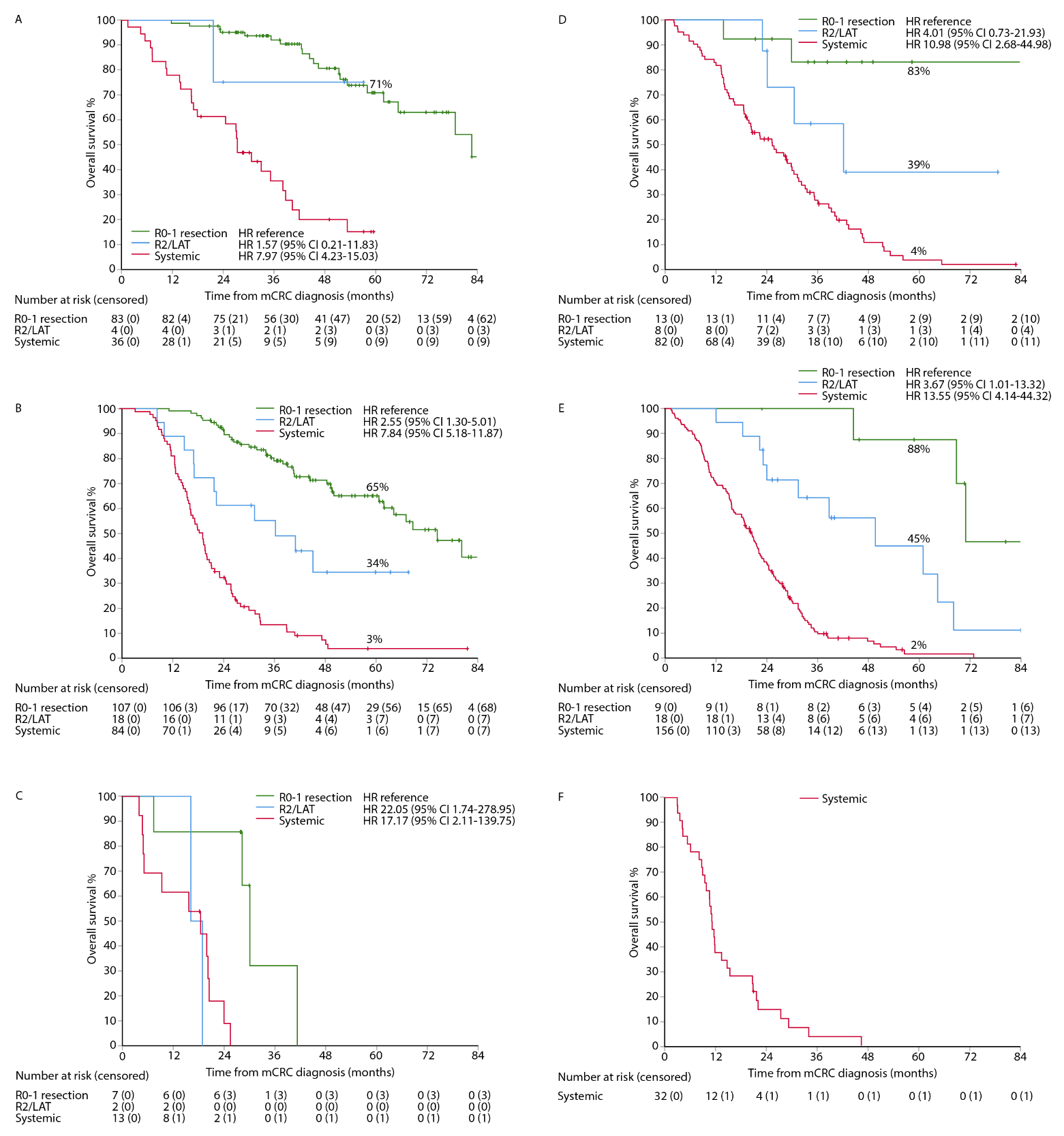


## Supplementary figure 4. 12-month conditional Landmark analysis of overall survival of patients according to resection status (R0/R1, R2 or LAT, systemic therapy only), mutations and extent of disease A. *RAS&BRAF*wt ‘liver-only’. B. *RAS*mt ‘liver-only’ C. *BRAF*mt ‘liver-only’ D. *RAS&BRAF*wt ‘liver and extrahepatic’ E. *RAS*mt ‘liver and extrahepatic’. F. *BRAF*mt ‘liver and extrahepatic’


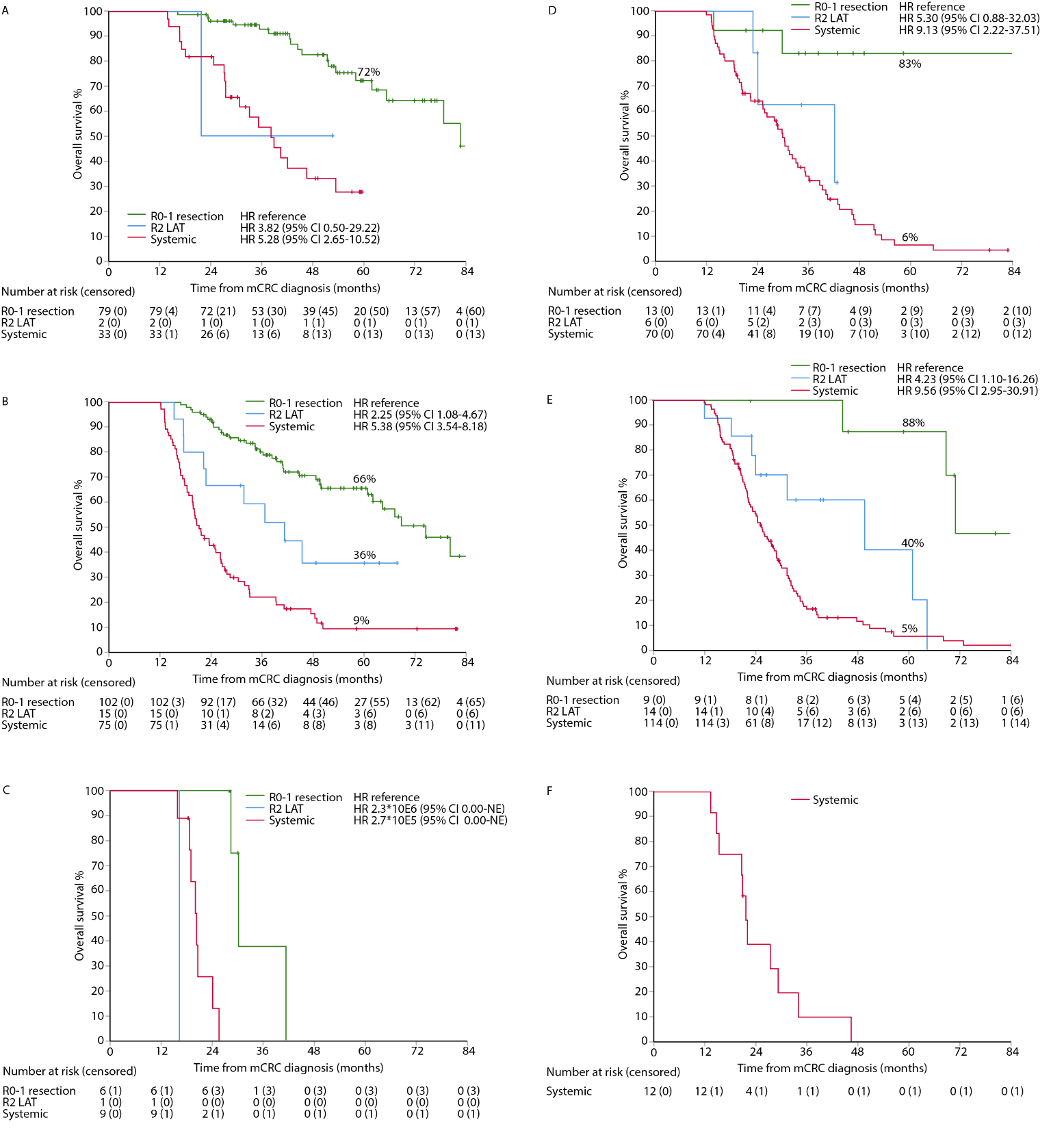


## Supplementary table 1. Patient demographics

|  |  | Total | | *RAS*&*BRAF*wt | | *RAS*mt | | *BRAF*mt | |
| --- | --- | --- | --- | --- | --- | --- | --- | --- | --- |
|  |  | 672 | 100 % | 226 | 100 % | 392 | 100 % | 54 | 100 % |
| Age | Median years (range) | 66 | (24-88) | 65 | (24-88) | 66 | (29-88) | 67 | (33-83) |
|  | ≤70 | 458 | 68 % | 156 | 69 % | 266 | 68 % | 36 | 67 % |
|  | >70 | 214 | 32 % | 70 | 31 % | 126 | 32 % | 18 | 33 % |
| Sex | Male | 428 | 64 % | 160 | 71 % | 244 | 62 % | 24 | 44 % |
|  | Female | 244 | 36 % | 66 | 29 %α | 148 | 38 %α | 30 | 56 %α |
| ECOG | PS 0 | 189 | 28 % | 62 | 27 % | 117 | 30 % | 10 | 19 % |
|  | PS 1 | 369 | 55 % | 127 | 56 % | 213 | 54 % | 29 | 54 % |
|  | PS 2-3 | 114 | 17 % | 37 | 16 % | 62 | 16 % | 15 | 28 % |
| Charlson comorbidity index | 0 | 517 | 77 % | 173 | 77 % | 304 | 78 % | 40 | 74 % |
|  | 1-2 | 149 | 22 % | 50 | 22 % | 85 | 22 % | 14 | 26 % |
|  | 3-5 | 6 | 1 % | 3 | 1 % | 3 | 1 % | 0 | 0 % |
| BMI | < 20 | 47 | 7 % | 15 | 7 % | 29 | 7 % | 3 | 6 % |
|  | 20-30 | 506 | 75 % | 173 | 77 % | 295 | 75 % | 38 | 70 % |
|  | ≥30 | 119 | 18 % | 38 | 17 % | 68 | 17 % | 13 | 24 % |
| Primary tumour location | Right colon | 185 | 28 % | 31 | 14 %β | 117 | 30 %β | 37 | 69 %β |
|  | Left colon | 264 | 39 % | 112 | 50 % | 141 | 36 % | 11 | 20 % |
|  | Rectum | 221 | 33 % | 82 | 36 % | 134 | 34 % | 5 | 9 % |
|  | Multiple | 2 | 0 % | 1 | 0 % | 0 | 0 % | 1 | 2 % |
| Histology | Adenocarcinoma | 618 | 92 % | 217 | 96 % | 358 | 91 % | 43 | 80 % |
|  | Signet ring or mucinous | 53 | 8 % | 8 | 4 %γ | 34 | 9 %γ | 11 | 20 %γ |
| Primary tumour resection | Upfront | 410 | 61 % | 139 | 62 % | 238 | 61 % | 33 | 61 % |
|  | During | 84 | 13 % | 34 | 15 % | 45 | 11 % | 5 | 9 % |
|  | No | 178 | 26 % | 53 | 23 % | 109 | 28 % | 16 | 30 % |
| Presentation of metastases | Synchronous* | 509 | 76 % | 158 | 70 % | 307 | 78 % | 44 | 81 % |
|  | Metachronous | 163 | 24 % | 68 | 30 % | 85 | 22 % | 10 | 19 % |
| Adjuvant chemotherapy for primary tumour | No adjuvant | 556 | 83 % | 175 | 77 %δ | 335 | 85 %δ | 46 | 85 %δ |
|  | Fluoropyrimidine | 42 | 6 % | 19 | 8 % | 20 | 5 % | 3 | 6 % |
|  | Oxaliplatin based | 74 | 11 % | 32 | 14 % | 37 | 9 % | 5 | 9 % |
| Radiotherapy for rectum | No | 600 | 89 % | 203 | 90 % | 345 | 88 % | 52 | 96 % |
|  | Preop 5x5 Gy | 32 | 5 % | 11 | 5 % | 21 | 5 % | 0 | 0 % |
|  | Chemoradiation | 27 | 4 % | 7 | 3 % | 19 | 5 % | 1 | 2 % |
|  | Palliative | 13 | 2 % | 5 | 2 % | 7 | 2 % | 1 | 2 % |
| 3 or more liver segments involved | No | 223 | 33 % | 79 | 35 % | 131 | 33 % | 13 | 24 % |
|  | Yes | 449 | 67 % | 147 | 65 % | 261 | 67 % | 41 | 76 % |
| Localisation of metastases | Unilateral | 433 | 64 % | 137 | 60 % | 256 | 65 % | 40 | 74 % |
|  | Bilateral | 239 | 36 % | 90 | 40 % | 135 | 35 % | 14 | 26 % |
| Metastatic sites | Liver-only | 354 | 53 % | 123 | 54 % | 209 | 53 % | 22 | 41 % |
|  | Liver and extrahepatic | 318 | 47 % | 103 | 46 % | 183 | 47 % | 32 | 59 % |
| Location of extrahepatic metastases | Lung | 178 | 26 % | 49 | 22 % | 114 | 29 % | 15 | 28 % |
|  | Lymph nodes | 149 | 22 % | 57 | 25 %η | 72 | 18 %η | 20 | 37 %η |
|  | Peritoneal | 60 | 9 % | 15 | 7 %ζ | 33 | 8 %ζ | 12 | 22 %ζ |
|  | Local relapse | 23 | 3 % | 11 | 5 % | 8 | 2 % | 4 | 7 % |
|  | Other | 60 | 7 % | 24 | 9 % | 32 | 6 % | 4 | 4 % |

*** Within 2 months from the diagnosis of primary tumour.

OR (CI95%) respectively for *RAS*&*BRAF*wt /*RAS*mt/*BRAF*mt:

α for female sex ref/1.5(1.0-2.1)/3.0(1.6-5.6), p=0.001 for whole comparison

β for more right-sided tumours than left-sided or rectal (multifocal excluded) ref/2.7(1.7-4.1)/14.5(7.2-29.1), p<0.001

γ for mucinous or signet cell histology ref/2.59(1.18-5.69)/6.97(2.65-18.35), p<0.001

δ for no adjuvant therapy after primary ref/1.7(1.0-2.7)/1.7(0.6-4.6), p=0.148

η for lymph node metastases more common ref/0.7(0.5-1.0)/1.7(0.9-3.3), p=0.003

ζ Peritoneal metastases more common ref/1.3(0.7-2.4)/4.0(1.8-9.2), p=0.001

## Supplementary table 2. Conversion rates for initially borderline resectable patients by primary tumour sidedness and systemic therapy regimens

|  |  | Total | | | | Right colon | | | | Left colon or rectum | | | |
| --- | --- | --- | --- | --- | --- | --- | --- | --- | --- | --- | --- | --- | --- |
|  | Conversion | Yes | | No | | Yes | | No | | Yes | | No | |
| ‘Liver-only’ mCRC* | | 78 | 100 % | 24 | 24 % | 13 | 52 % | 12 | 48 % | 65 | 84 % | 12 | 16 % |
|  | Combination chemotherapy with cetuximab or panitumumab | 17 | 85 % | 3 | 15 % | 0 | 0 % | 1 | 100 % | 17 | 90 % | 2 | 10 % |
|  | Combination chemotherapy with bevacizumab | 48 | 77 % | 14 | 23 % | 11 | 55 % | 9 | 45 % | 37 | 88 % | 5 | 12 % |
|  | 1-2 drugs (chemotherapy and/or biologic) | 13 | 65 % | 7 | 35 % | 2 | 50 % | 2 | 50 % | 11 | 69 % | 5 | 31 % |
|  | *RAS*&*BRAF* wild type | 30 | 79 % | 8 | 21 % | 0 | 0 % | 1 | 100 % | 30 | 81 % | 7 | 19 % |
|  | *RAS* mutated type | 44 | 81 % | 10 | 19 % | 12 | 71 % | 5 | 30 % | 32 | 86 % | 5 | 14 % |
|  | *BRAF* mutated type | 4 | 40 % | 6 | 60 % | 1 | 14 % | 6 | 86 % | 3 | 100 % | 0 | 0 % |
| ‘Liver and extrahepatic’ mCRC | | 22 | 63 % | 13 | 37 | 7 | 64 % | 4 | 36 % | 15 | 63 % | 9 | 37 % |
|  | Combination chemotherapy with cetuximab or panitumumab | 4 | 67 % | 2 | 33 % | 0 | 0 % | 0 | 0 % | 4 | 67 % | 2 | 33 % |
|  | Combination chemotherapy with bevacizumab | 14 | 61 % | 9 | 39 % | 5 | 63 % | 3 | 37 % | 9 | 60 % | 6 | 40 % |
|  | 1-2 drugs (chemotherapy and/or biologic) | 4 | 67 % | 2 | 33 % | 2 | 67 % | 1 | 33 % | 2 | 67 % | 1 | 33 % |
|  | *RAS*&*BRAF* wild type | 7 | 54 % | 6 | 46 % | 6 | 50 % | 6 | 50 % | 1 | 100 % | 0 | 0 % |
|  | *RAS* mutated type | 15 | 63 % | 9 | 38 % | 9 | 60 % | 6 | 40 % | 6 | 67 % | 3 | 33 % |
|  | *BRAF* mutated type | 0 | 0 % | 1 | 100 % | 0 | 0 % | 0 | 0 % | 0 | 0 % | 1 | 100 % |

* mCRC Metastatic colorectal cancer.

## Supplementary table 3. Reasons for not resecting patients who were centrally assessed as technically resectable

|  |  | Total | | *RAS&BRAF* wt | | *RAS* mt | | *BRAF* mt | |
| --- | --- | --- | --- | --- | --- | --- | --- | --- | --- |
| ‘Liver-only’ mCRC* | | 19 | 100 % | 3 | 100 % | 15 | 100 % | 1 | 100 % |
|  | Comorbidities | 5 | 26 % | 0 | 0 % | 5 | 33 % | 0 | 0 % |
|  | Progressive disease | 12 | 63 % | 2 | 67 % | 9 | 60 % | 1 | 100 % |
|  | Inoperabile in exploration | 2 | 11 % | 1 | 33 % | 1 | 7 % | 0 | 0 % |
| ‘Liver and extrahepatic’ mCRC | | 9 | 100 % | 2 | 100 % | 7 | 100 % | 0 | 0 % |
|  | Comorbidities | 3 | 33 % | 1 | 50 % | 2 | 29 % | 0 | 0 % |
|  | Progressive disease | 5 | 56 % | 1 | 50 % | 4 | 57 % | 0 | 0 % |
|  | Inoperabile in exploration | 1 | 11 % | 0 | 0 % | 1 | 14 % | 0 | 0 % |

* mCRC Metastatic colorectal cancer.

**References**

1. Osterlund P, Salminen T, Soveri LM, Kallio R, Kellokumpu I, Lamminmaki A, et al. Repeated centralized multidisciplinary team assessment of resectability, clinical behavior, and outcomes in 1086 Finnish metastatic colorectal cancer patients (RAXO): A nationwide prospective intervention study. Lancet Reg Health Eur. 2021;3.

2. Isoniemi H, Uutela A, Nordin A, Lantto E, Kellokumpu I, Ovissi A, et al. Centralized repeated resectability assessment of patients with colorectal liver metastases during first-line treatment: prospective study. Br J Surg. 2021.

3. Uutela A, Osterlund E, Halonen P, Kallio R, Algars A, Salminen T, et al. Resectability, conversion, metastasectomy and outcome according to RAS and BRAF status for metastatic colorectal cancer in the prospective RAXO study. Br J Cancer. 2022.

4. Cervantes A, Adam R, Rosello S, Arnold D, Normanno N, Taieb J, et al. Metastatic colorectal cancer: ESMO Clinical Practice Guideline for diagnosis, treatment and follow-up (Journal Pre-proof). Ann Oncol. 2022:doi.org/10.1016/j.annonc.2022.10.003.

5. Benson AB, Venook AP, Al-Hawary MM, Arain MA, Chen YJ, Ciombor KK, et al. Colon Cancer, Version 2.2021, NCCN Clinical Practice Guidelines in Oncology. J Natl Compr Canc Netw. 2021;19(3):329-59.

6. Benson AB, Venook AP, Al-Hawary MM, Arain MA, Chen YJ, Ciombor KK, et al. Rectal Cancer, Version 2.2021, NCCN Clinical Practice Guidelines in Oncology. 2021.

7. Nordlinger B, Sorbye H, Glimelius B, Poston GJ, Schlag PM, Rougier P, et al. Perioperative FOLFOX4 chemotherapy and surgery versus surgery alone for resectable liver metastases from colorectal cancer (EORTC 40983): long-term results of a randomised, controlled, phase 3 trial. Lancet Oncol. 2013;14(12):1208-15.

8. Custodio A, Feliu J. Prognostic and predictive biomarkers for epidermal growth factor receptor-targeted therapy in colorectal cancer: beyond KRAS mutations. Crit Rev Oncol Hematol. 2013;85(1):45-81.
